# Enhancing genomic mutation data storage optimization based on the compression of asymmetry of sparsity

**DOI:** 10.3389/fgene.2023.1213907

**Published:** 2023-06-01

**Authors:** Youde Ding, Yuan Liao, Ji He, Jianfeng Ma, Xu Wei, Xuemei Liu, Guiying Zhang, Jing Wang

**Affiliations:** ^1^ The Sixth Affiliated Hospital of Guangzhou Medical University, Qingyuan People’s Hospital, Qingyuan, China; ^2^ School of Biomedical Engineering, Guangzhou Medical University, Guangzhou, China

**Keywords:** genomic, sparse, compression, single-nucleotide variation, copy number variation

## Abstract

**Background:** With the rapid development of high-throughput sequencing technology and the explosive growth of genomic data, storing, transmitting and processing massive amounts of data has become a new challenge. How to achieve fast lossless compression and decompression according to the characteristics of the data to speed up data transmission and processing requires research on relevant compression algorithms.

**Methods:** In this paper, a compression algorithm for sparse asymmetric gene mutations (CA_SAGM) based on the characteristics of sparse genomic mutation data was proposed. The data was first sorted on a row-first basis so that neighboring non-zero elements were as close as possible to each other. The data were then renumbered using the reverse Cuthill-Mckee sorting technique. Finally the data were compressed into sparse row format (CSR) and stored. We had analyzed and compared the results of the CA_SAGM, coordinate format (COO) and compressed sparse column format (CSC) algorithms for sparse asymmetric genomic data. Nine types of single-nucleotide variation (SNV) data and six types of copy number variation (CNV) data from the TCGA database were used as the subjects of this study. Compression and decompression time, compression and decompression rate, compression memory and compression ratio were used as evaluation metrics. The correlation between each metric and the basic characteristics of the original data was further investigated.

**Results:** The experimental results showed that the COO method had the shortest compression time, the fastest compression rate and the largest compression ratio, and had the best compression performance. CSC compression performance was the worst, and CA_SAGM compression performance was between the two. When decompressing the data, CA_SAGM performed the best, with the shortest decompression time and the fastest decompression rate. COO decompression performance was the worst. With increasing sparsity, the COO, CSC and CA_SAGM algorithms all exhibited longer compression and decompression times, lower compression and decompression rates, larger compression memory and lower compression ratios. When the sparsity was large, the compression memory and compression ratio of the three algorithms showed no difference characteristics, but the rest of the indexes were still different.

**Conclusion:** CA_SAGM was an efficient compression algorithm that combines compression and decompression performance for sparse genomic mutation data.

## 1 Introduction

Genes are one of the basic units of life and are of irreplaceable importance in the fields of understanding life phenomena, exploring the laws of biological evolution, and preventing and controlling human diseases (Tu et al., 2006; Oh et al., 2012). Gene sequences are the carriers of biological genetic information, and the biological properties of all organisms are related to genes (Mota and Franke, 2020). Due to the enormous usefulness of genetic data and the reduced cost of sequencing, many countries and organizations have initiated various genetic engineering projects, such as the Personal Genome Project (Ball et al., 2012) and the Bio Genome Project (Lewin et al., 2018). The rapid growth of genetic data can provide a significant boost to the life sciences. A rich gene pool can be very beneficial to the study of certain types of diseases, providing a new breakthrough to promote precision medicine and help solve medical problems (Janssen et al., 2011; Chen et al., 2020; Garand et al., 2020).

However, the growth of genetic data has now greatly outpaced the growth of storage and transmission bandwidth, posing significant storage and transmission challenges (Xi et al., 2023a). The Human Genome Project (Cavalli-Sforza, 2005; Boeke et al., 2016) and the 1000 Genomes Project (Belsare et al., 2019; Fairley et al., 2020), for example, generate huge amounts of data, tens of terabytes or even more. Thus, issues related to genetic data compression have become a hot topic and focus of research in recent years. Genomic mutation data contain a large amount of genetic variation information that can be used to resolve the functional and phenotypic effects of genetic variants, which is of great value for human evolutionary genetic and medical research. Comparative databases (such as dbSNP and ClinVar) allow sequencing and differential analysis of genes in individuals or populations of species. Genetic information such as single-nucleotide variation (SNV), insertion deletion (InDel), structural variation (SV) and copy number variation (CNV) can be used to develop molecular markers and create databases of genetic polymorphisms. Cross-species genome alignment methods provide genomic context for the identification of annotated gene regions for variation across species (Samaha et al., 2021). In recent years, many researchers have developed a variety of rapid detection methods or tools for CNV (Huang et al., 2021; Lavrichenko et al., 2021; Kim et al., 2022) and SNV (van der Borght et al., 2015; Schnepp et al., 2019; Li et al., 2022). However, variant genomic mutation data are often sparse data formats that are difficult to apply with traditional compression methods.

Traditional compression algorithms generally reduce the storage space of data by encoding it, such as Huffman coding (Moffat, 2019), Lempel-Ziv-Welch coding (Fira and Goras, 2008; Naqvi et al., 2011), etc. These algorithms are designed based on the assumption that there is a large amount of repetitive information in the data. But for sparse data, there is less redundancy in the information present in the data, making it difficult to compress effectively. The operations in turn waste a lot of time performing invalid operations with zero elements. As a result, traditional algorithms such as gzip, bzip2, lzo, snappy, etc. Are memory wasting and inefficient. As a result, compressed storage methods for sparse genes, a special form of data, have received increasing attention from researchers (Shekaramiz et al., 2019; Yao et al., 2019; Li et al., 2021; Wang et al., 2022). Although there are some sparse compression methods available, such as coordinate format (COO) and compressed sparse column format (CSC) compression (Park et al., 2020), they suffer from different drawbacks. Some are difficult to operate and cannot perform matrix operations, while others have problems such as slow inner product operations and slow row/column slicing operations, so none are particularly desirable either.

In this paper, based on the sparse asymmetry of variant genomic data, we propose a method for lossless compression of genomic mutation data called CA_SAGM. Preprocessing steps such as prioritization and reverse Cuthill-Mckee (RCM) sorting are performed on the data to greatly reduce the bandwidth of the matrix, so that the scattered non-zero elements all converge towards the diagonal. The data is then compressed sparse row format (CSR) (Koza et al., 2014; Chen et al., 2018; Xing et al., 2022) and stored. This method can theoretically optimize the efficiency and quality of the rearranged data, saving processing time and memory requirements. This study shows that CA_SAGM exhibits higher compression performance and best decompression performance for sparse genomic data compared to COO and CSC. From a combination of several evaluation metrics such as compression and decompression time, compression and decompression rate, compression size and compression ratio, the CA_SAGM method performs the best and outperforms the rest of the methods. It is confirmed that the CA_SAGM method has fast and efficient compression and decompression performance for sparse genomic data, has good applicability and can be further extended to other similar data.

## 2 Materials and methods

Both SNV and CNV are common formats for genomic mutation data storage. SNV is a single nucleotide mutation resulting in a deletion, insertion or substitution in a normal human gene. Large-scale tumor sequencing studies have shown that most cancers are caused by SNV (Macintyre et al., 2016). DNA copy number variation is a structural form of genomic variation (Medvedev et al., 2009; Stankiewicz and Lupski, 2010). Many studies have shown that CNVs are associated with complex diseases such as autism, schizophrenia, Alzheimer’s disease, and cancer. In recent years, there have been a large number of studies on SNVs and CNVs (Jugas et al., 2021; Prashant et al., 2021; Ladeira et al., 2022; Lee et al., 2022; Li et al., 2022; Zheng, 2022).

### 2.1 Materials

In this paper, SNV data for nine different diseases and CNV data for six different diseases were selected from the TCGA database (The ICGC/TCGA Pan-Cancer Analysis of Whole Genomes Consortium, 2020), all data are level3. SNVs or mutations are less common than other variants and mutations and cannot be observed in the diversity of the genome (Press et al., 2019). It is a single-nucleotide variation without any frequency restriction and may arise in somatic cells. The number and type of SNVs and other characteristics can reflect the genetic diversity, evolutionary history and other information of a species. SNVs also play an important role in the occurrence and development of human diseases (Xi et al., 2020a; Xi et al., 2023b). For example, some SNVs may cause gene mutations and affect protein structure and function, leading to the development of diseases; SNV-based research also helps to find susceptibility genes for diseases and develop corresponding drug targets, etc. SNV data are from brain tumor, acute myeloid leukemia, thyroid cancer, prostate cancer, ovarian cancer, breast cancer, bladder cancer, renal clear cell carcinoma and colorectal cancer.

CNV, or copy number variation, is caused by rearrangements in the genome. It generally refers to an increase or decrease in the copy number of a large segment of the genome. It is mainly manifested as deletions and duplications at the sub-microscopic level. CNV is an important genetic basis for individual differences and is widely distributed in the human genome (Xi and Li, 2016; Xi et al., 2020b). The CNV data are more complex than the SNV data, with larger data sets, higher numbers of non-zeros and higher densities. CNV data were obtained from acute spinal leukemia, thyroid cancer, prostate cancer, bladder cancer, renal clear cell carcinoma and colorectal cancer. The basic characteristics of SNV and CNV data were analyzed in detail, including Data set size (n), non-zero number (n), sparsity (%), rows (n), rows/columns (%), file size (K), L1-norm, L2-norm and Rank. They are shown in Tables 1, 2 respectively.

**TABLE 1:** Raw SNV data benchmark results.

SNV data	Data set size(n)	Non-zero number(n)	Sparsity (%)	Rows (n)	Rows/columns (%)	File size (K)	L1-norm	L2-norm	Rank
Brain tumor	1006707	395	0.039	83	0.684	2	38	10.167	66
Acute myelogenous leukemia	2377284	1683	0.071	196	1.616	7	56	11.417	187
Thyroid carcinoma	4863729	4780	0.098	401	3.306	17	241	16.173	400
Prostate cancer	4038957	9004	0.223	333	2.745	27	52	29.835	332
Ovarian cancer	3832764	12873	0.336	316	2.605	35	312	22.599	316
Breast cancer	6202131	20287	0.327	507	4.145	52	208	24.876	507
Bladder cancer	1576770	25368	1.609	130	1.072	53	167	52.160	130
Clear cell carcinoma of kidney	5142696	24023	0.470	424	3.496	60	274	30.251	424
Colorectal cancer	2716896	48038	1.768	224	1.847	91	415	294.987	224
mean	3528659.33	16272.33	0.55	290.44	2.39	38.22	195.89	54.72	287.33
sd	1733587.32	15121.90	0.62	142.10	1.10	28.60	130.29	90.98	145.89

Where n represents the number of data, % represents the percentage and k represents kilobytes.

**TABLE 2:** Raw CNV data benchmark results.

CNV data	Data set size(n)	Non-zero number(n)	Non-negative ratio(%)	Sparsity (%)	Rows (n)	Rows/columns (%)	File size (K)	L1-norm	L2-norm	Rank
Acute myelogenous leukemia	2316639	81104	48.12	3.50	191	1.574738	92	34	143.673	157
Thyroid carcinoma	6015984	189060	58.81	3.14	496	4.089373	175	92	224.882	279
Prostate cancer	4038957	556304	38.74	13.77	333	2.745486	812	259	372.086	325
Colorectal cancer	3117153	753833	54.11	24.18	257	2.118889	910	224	492.879	253
Bladder cancer	1552512	780530	53.59	50.28	128	1.055322	800	144	435.185	127
Clear cell carcinoma of kidney	5288244	1196243	50.32	22.62	436	3.59469	1489	420	653.243	433
mean	3721581.50	592845.67	50.62	19.58	306.83	2.530	713.00	195.50	386.99	262.33
sd	1724152.03	412671.39	6.86	17.52	142.15	1.171994	516.42	137.62	184.46	112.1

Where n represents the number of data, % represents the percentage and k represents kilobytes.

### 2.2 Methods

#### 2.2.1 Compression algorithm

COO and CSC are two common compression methods for sparse data. COO uses a triplet to store information about the non-zero elements of the matrix, storing the row subscripts, column subscripts and values of the non-zero elements respectively. The non-zero elements are found by traversing the rows and columns and storing the corresponding number of rows, columns and values in the corresponding arrays. Let A ∈ Rm×*n* be a sparse matrix where the number of non-zero elements. Using the COO storage method, A can be stored as three vectors (I, J, V). Where I and J store the coordinates of the rows and columns of the non-zero elements respectively, and V stores the values of the non-zero elements. Examples of mathematical formulas are as follows:

$$A=\left[\begin{array}{ccc} \partial_{00} & 0 & \partial_{02}\\ 0 & 0 & \partial_{12}\\ \partial_{20} & \partial_{21} & \partial_{22} \end{array}\right]\Rightarrow I=\left[\begin{array}{l} 0\\ 0\\ 1\\ 2\\ 2\\ 2 \end{array}\right],J=\left[\begin{array}{l} 0\\ 2\\ 2\\ 0\\ 1\\ 2 \end{array}\right],V=\left[\begin{array}{l} \partial_{00}\\ \partial_{02}\\ \partial_{12}\\ \partial_{20}\\ \partial_{21}\\ \partial_{22} \end{array}\right]$$ (1)

Data can be converted to other storage formats by COO method quickly and easily, and data can be quickly converted with compressed sparse row format (CSR)/CSC formats and can be repeatedly indexed. However, the COO format is almost impossible to manipulate or matrix-operate except by converting it to other formats.

The CSC is compressed and stored according to the principle of data column precedence. The matrix is determined by the row indexes of non-zero elements, index pointers, and non-zero data. Suppose an m × *n* sparse matrix, with A_ij_ denoting the elements of row i and column j. CSC can store A as three vectors (in_dices, indptr and value). Where in_dices is the row index of the non-zero elements, indptr is an array of index pointers and value is the non-zero data in the matrix. The steps are as follows:

1. Get the row index of the non-zero element in column i according to indices [indptr[i]: indptr[i+1]].
2. Get the number of non-zero elements in column i according to [indptr[i]: indptr[i+1]].
3. The column index and row index are obtained and the corresponding data is stored in: value [indptr[i]: indptr[i+1]]. The following mathematical formula is an example:

$$A=\left[\begin{array}{ccc} \partial_{00} & 0 & \partial_{02}\\ 0 & 0 & \partial_{12}\\ \partial_{20} & \partial_{21} & \partial_{22} \end{array}\right]\Rightarrow indptr=\left[\begin{array}{l} 0\\ 2\\ 3\\ 6 \end{array}\right],in\_dices=\left[\begin{array}{l} 0\\ 2\\ 2\\ 0\\ 1\\ 2 \end{array}\right],value=\left[\begin{array}{l} \partial_{00}\\ \partial_{20}\\ \partial_{21}\\ \partial_{02}\\ \partial_{12}\\ \partial_{22} \end{array}\right]$$ (2)

The CSC data format performs efficient column slicing, but the inner matrix product and row slicing operations are relatively slow.

#### 2.2.2 CA_SAGM algorithm

CA_SAGM is an optimization algorithm based on compressed sparse row format, which is implemented by optimizing the matrix ordering for the characteristics of variable genomic data. The process is as follows: first, the variant genomic data is sorted by row-major order so that adjacent non-zero elements are also physically stored as close as possible to each other. Then, the reverse Cuthill-McKee sorting algorithm is used to renumber the rows and columns of the data according to the sorting results. Finally, using the new row and column numbering, the sparse matrix is CSR compressed and stored in a file.

Reverse Cuthill-Mckee sorting is an algorithm that can be used to optimize the storage of sparse matrices by rearranging the rows and columns of a sparse matrix so that the matrix has a smaller bandwidth. Bandwidth is understood to be the widest diagonal distance between the non-zero elements of a matrix and has a significant impact on the efficiency of computational operations such as matrix multiplication. The basic idea of the RCM sorting algorithm is to reduce the bandwidth of a matrix by arranging interconnected points as close to each other as possible. The sparse matrix is first transformed into an undirected graph, and then this graph is traversed and pruned as a way to determine the new order of nodes, which in turn leads to the rearranged matrix. Specifically, when a node is processed, the traversal of that branch is stopped if the number of remaining nodes is not sufficient to cause a smaller bandwidth to the already traversed nodes. In addition, the RCM sorting algorithm can also use other heuristic rules such as degree sorting and greedy strategy to further improve the efficiency and quality of matrix reordering. The main ideological steps of the RCM algorithm are as follows:

1. Select a starting point and mark it as a visited node.
2. Sort the nodes adjacent to this starting point in order of traversal distance from closest to farthest.
3. Recursively executes steps 1 and 2 for the sorted neighboring nodes.
4. When all adjacent nodes have been traversed, return to the previous level of nodes and continue until the last level has been traversed.
5. For all unvisited nodes, sort the nodes according to the depth-first traversal method, again prioritizing the nodes adjacent to the visited nodes until all nodes have been traversed.

Sparse genomic matrix data has a large bandwidth due to the dispersed arrangement of non-zero elements. With the use of reverse Cuthill-Mckee matrix bandwidth compression, the bandwidth of the matrix is greatly reduced, and the scattered non-zero elements all converge towards the diagonal, which greatly improves computational efficiency and reduces memory requirements according to the relationship between computational complexity of lower-upper (LU) decomposition and memory requirements and bandwidth, which is followed by LU decomposition after RCM pre-processing. For most sparse matrix problems, due to the small number of elements being sorted, RCM has proven to be a more efficient algorithm in practice, as neither quick sort nor merge sort is as fast. It performs as fast as traditional execution, but with no reduction in speed for problems with a high number of nodes. The steps of the reverse Cuthill-Mckee algorithm are as follows:

1. Instantiate an empty queue Q for the alignment of the object R.
2. Find the object with the smallest degree whose index has not been added to R. Assume that the object corresponding to row p has been identified as the object with the smallest degree. Add p to R. (The degree of a node is defined as the sum of the non-diagonal elements in the corresponding row.)
3. Add the index to R, and add all neighbors of the corresponding object at the index, in increasing order to Q. Neighbors are nodes with non-zero values between them.
4. Extract the first node in Q, e.g., C. Insert C into R if it has not already been inserted, then add Q’s C neighbors to Q in increasing order.
5. If Q is not empty, repeat step4.
6. If Q is empty, but there are objects in the matrix that are not yet included in R, start again from Step2.
7. Until all objects are contained in R terminate the algorithm.

### 2.3 Performance evaluation metrics

A number of metrics were used to evaluate the compression and decompression performance between COO, CSC and CA_SAGM. Compression time (CT, Milliseconds or Seconds), compression rate (CR, Megabytes/Second), compression memory (CM, Kilobytes or Megabytes), compression ratio (CRO), decompression time (DCT, Milliseconds) and decompression rate (DCR, Megabytes/Second) are included. These parameters are calculated in Eqs 3–8.

CT=Compression end time−Compression start time (3)

DCT=Decompression end time−Decompression start time (4)

CR=Compression size / CT (5)

DCR=Decompression size / DCT (6)

CM=Memory size after compression (7)

CRO=Pre−compressed memory / post−compressed memory (8)

The above metrics allow the compression algorithms to be evaluated in terms of the speed at which the data is compressed/decompressed for work, the amount of data, the memory space occupied and other different aspects. In general, shorter CT and DCT, faster CR and DCR, smaller CM and larger CRO represent better compression and decompression performance. And, we performed a statistical analysis of the experimental results. However, we can also evaluate algorithms based on different data, different usage scenarios and requirements. Different compression algorithms will perform differently in these performance metrics, users will need to choose the right algorithm for their specific scenario and needs.

## 3 Experiments and results

In order to objectively compare the performance metrics of the different algorithms, all experiments were conducted in the same environmental configuration. The system configuration used in this study is Windows 10 (Microsoft Corporation, United States), CPU: Inter(R) Core(TM) I5-10500, 3.10 GHz; RAM: 8 G. The compression algorithm processing software is MATLAB R2022a (Mathworks. United States). And the statistical analysis software is IBM SPSS Statistics 26 (IBM Corp. United States). No other applications were run during any of the programs to ensure a consistent working environment.

### 3.1 SNV data compression performance

#### 3.1.1 Comparison of SNV data compression algorithms

**FIGURE 1:**
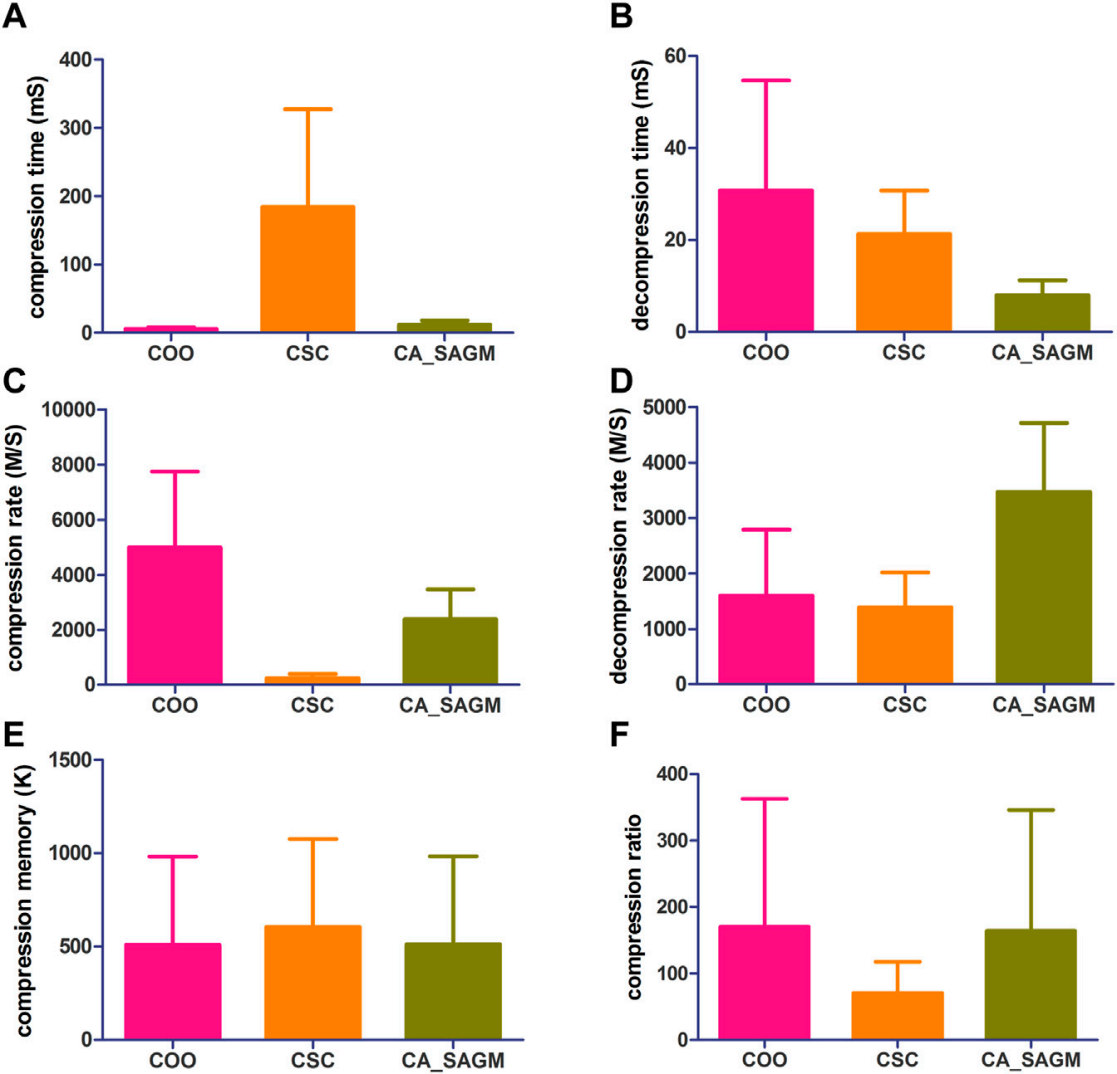
Compares the compression and decompression metrics of COO, CSC and CA_SAGM for SNV. Where **(A)** stands for compression time, **(B)** for decompression time, **(C)** for compression speed, **(D)** for decompression speed, **(E)** for compressed memory and **(F)** for compression ratio.

The general process of processing SNV data includes data read-in, pre-processing, compression and storage. The original SNV data is read in and tested for basic characteristics, including data set size (n), non-zero number (n), sparsity (%), rows (n), rows/columns (%), file size (K), L1-norm, L2-norm and rank. First, the SNV data runs the COO and CSC programs separately. The sparse data matrix was then preprocessed by row-first sorting and RCM sorting successively. Next, SNV data were run through CA_SAGM compression programs. Compression time, decompression time, compression rate, decompression rate, compression memory and compression ratio are respectively obtained by the three methods. The results are shown in Figure 1. Finally, the compressed data were stored in a suitable location. The experimental results were in mean ± SD (Standard deviation, SD) format, and were analyzed by comparing the evaluation indexes among different algorithms and using statistical methods.

As can be seen from the Figure 1, the COO algorithm has the shortest CT (5.91 ± 2.42 vs. 184.61 ± 142.89 vs. 12.25 ± 5.81), the largest CR (4989.43 ± 2753.14 vs. 238.85 ± 153.2 vs. 2377.17 ± 1093.17), the smallest CM (509.53 ± 472.56 vs. 604.55 ± 472.59 vs. 511.97 ± 472.65), CRO was the largest (170.31 ± 192.38 vs. 70.8 ± 46.65 vs. 164.48 ± 181.78), compression performance was the best. However, decompression took the longest to recover the original data (30.76 ± 23.89 vs. 21.33 ± 9.42 vs. 7.96 ± 3.32) and had a smaller decompression rate (1596.72 ± 1187.87 vs. 1389.44 ± 629.08 vs. 3467.85 ± 1246.34). The performance of CSC was the opposite of COO, CT was the longest (184.61 ± 142.89), CR was the lowest (238.85 ± 153.2), CM was the largest (604.55 ± 472.59) and CRO was the smallest (70.8 ± 46.65). The decompression performance of CSC is between COO and CA_SAGM, with DCT and DCR both performing in the middle. In addition, CA_SAGM has the best decompression performance, with the shortest DCT (7.96 ± 3.32) and the largest DCR (3467.85 ± 1246.34). If the overall total time of compression and decompression time, the average rate of compression rate and decompression rate are considered, it is clear that the CA_SAGM algorithm has the shortest total time and the largest average rate.

A paired sample *t*-test was used to assess whether there were differences in the same metrics between any two algorithms. The results show that there is a significant difference (*p* < 0.05) between any two algorithms for almost all metrics: compression time (COO to CSC: 0.005; COO to CA_SAGM: 0.001; CA_SAGM to CSC: 0.006), decompression time (COO to CSC: 0.111; COO to CA_SAGM: 0.013; CA_SAGM to CSC: 0.000), compression rate (COO to CSC: 0.001; COO to CA_SAGM: 0.003; CA_SAGM to CSC: 0.000), decompression rate (COO to CSC: 0.493; COO to CA_SAGM: 0.001; CA_SAGM to CSC: 0.000), compression memory (COO to CSC: 0.000; COO to CA_SAGM: 0.000; CA_SAGM to CSC: 0.000), compression ratio (COO to CSC: 0.003; COO to CA_SAGM: 0.000; CA_SAGM to CSC: 0.003). There is little difference between COO and CSC in terms of compression time and decompression speed.

#### 3.1.2 Correlation analysis of SNV data

We used spearman correlation analysis to investigate whether the compression and decompression performance was correlated with the basic characteristics of the original SNV data. Table 3 shows that the compression time, decompression time, compression rate, decompression rate, compression memory and compression ratio are all correlated with the non-zero number of the original data, sparsity, file size, L1-norm and L2-norm. There was a strong correlation between sparsity and the non-zero number of raw data (*p* = 0.983), file size (*p* = 0.967), L1-norm (*p* = 0.983) and L2-norm (*p* = 0.983).

**TABLE 3:** Spearman correlation analysis between compression and decompression metrics of COO, CSC and CA_SAGM algorithms for SNV data and basic characteristics of the original data.

Index	Data set size(n)	Non-zero number(n)	Sparsity (%)	Rows (n)	Row/column (%)	File size (K)	L1-norm	L2-norm	Rank
1_CT	0.167	.983**	.967**	0.167	0.167	.967**	0.617	.933**	0.167
2_CT	0.233	1.000**	.983**	0.233	0.233	.983**	.667*	.950**	0.233
3_CT	0.467	.933**	.883**	0.467	0.467	.950**	.700*	.850**	0.467
1_DCT	0.333	.983**	.967**	0.333	0.333	1.000**	.717*	.933**	0.333
2_DCT	0.45	.950**	.917**	0.45	0.45	.983**	.733*	.883**	0.45
3_DCT	.717*	.750*	.717*	.717*	.717*	.833**	.667*	.683*	.717*
1_CM	0.233	1.000**	.983**	0.233	0.233	.983**	.667*	.950**	0.233
2_CM	0.233	1.000**	.983**	0.233	0.233	.983**	.667*	.950**	0.233
3_CM	0.233	1.000**	.983**	0.233	0.233	.983**	.667*	.950**	0.233
1_CR	.833**	−0.3	−0.35	.833**	.833**	−0.217	0.033	−0.283	.833**
2_CR	−0.033	−.933**	−.950**	−0.033	−0.033	−.917**	−0.6	−.883**	−0.033
3_CR	0.45	−0.55	−0.517	0.45	0.45	−0.517	−0.133	−0.483	0.45
1_DCR	−0.167	−.983**	−1.000**	−0.167	−0.167	−.967**	−.717*	−.933**	−0.167
2_DCR	0.333	−.733*	−.783*	0.333	0.333	−.700*	−0.333	−.717*	0.333
3_DCR	0.467	−0.6	−0.617	0.467	0.467	−0.55	−0.167	−0.55	0.467
1_CRO	0.167	.983**	1.000**	0.167	0.167	.967**	.717*	.933**	0.167
2_CRO	−0.117	.867**	.883**	−0.117	−0.117	.850**	0.533	.783*	−0.117
3_CRO	0.167	.983**	1.000**	0.167	0.167	.967**	.717*	.933**	0.167

Where 1_ represents the COO compression algorithm, 2_ represents the CSC compression algorithm and 3_ represents the CA_SAGM compression algorithm. ** At level 0.01, the correlation was significant.* At level 0.05, the correlation was significant.

**FIGURE 2:**
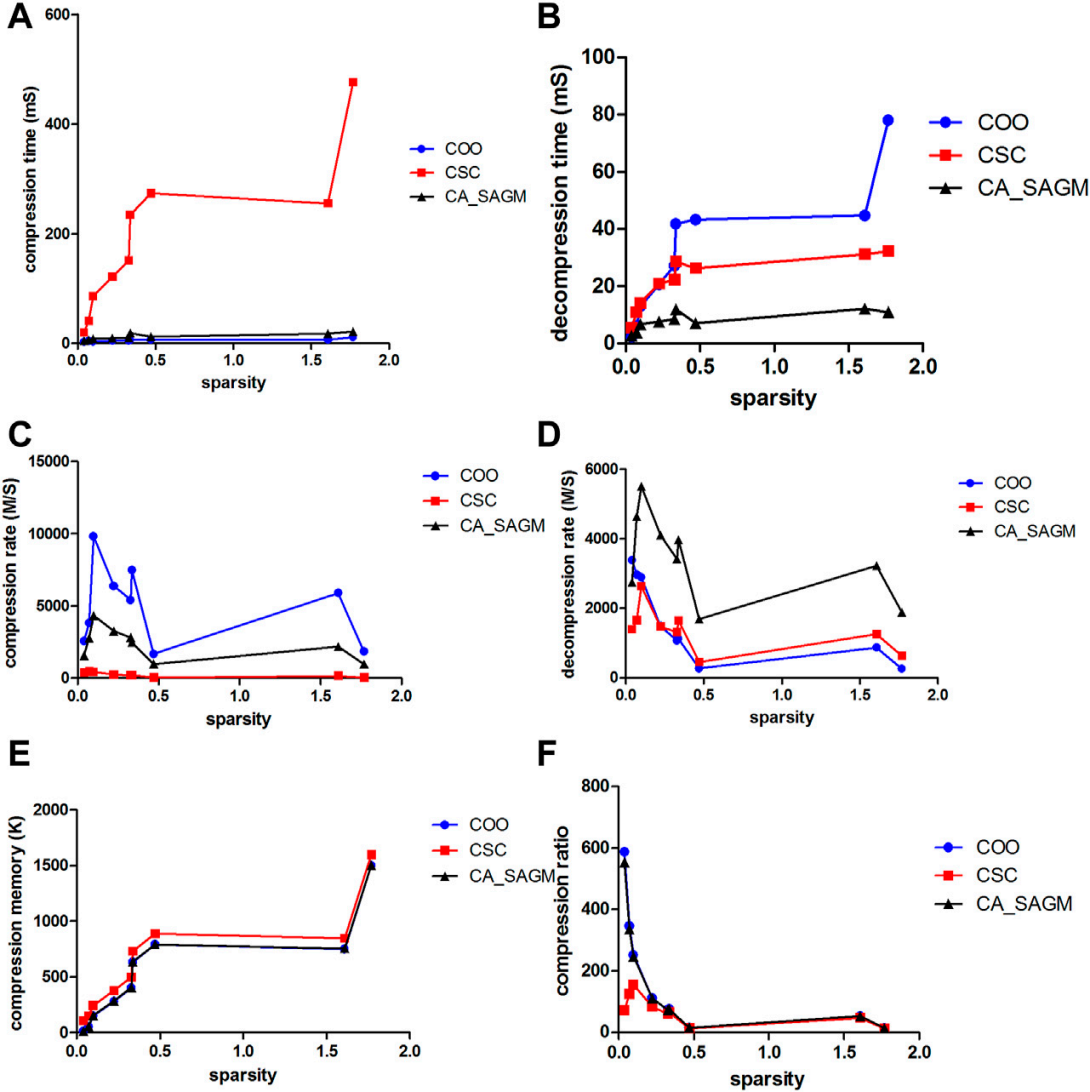
Curves of compression and decompression metrics vs. sparsity variation for COO, CSC and CA_SAGM for SNV. Where **(A)** stands for compression time, **(B)** for decompression time, **(C)** for compression speed, **(D)** for decompression speed, **(E)** for compressed memory and **(F)** for compression ratio.

As sparsity is easy to calculate and obtain, we further analyzed the effect of sparsity on the SNV data, as shown in Figure 2. As can be seen from the figure, CSC compression performance performs the worst, with the longest CT, the smallest CR and the smallest CRO. Both COO and CA_SAGM show better compression characteristics, with shorter CT and larger CR. In terms of decompression, COO performs the worst, with the longest DCT and smallest DCR. CA_SAGM performs the best, with the shortest DCT and largest DCR, CSC performs in the middle. The difference between the compression & decompression performance of COO, CSC and CA_SAGM is small when the sparsity is close to 0. As the data sparsity increases (but the sparsity is still small, <2%), the compression & decompression time tends to become larger, the compression and decompression rate tends to decrease, and the compression ratio also tends to decrease. The difference in compression and decompression times between algorithms increases with sparsity.

### 3.2 CNV data compression performance

#### 3.2.1 Comparison of CNV data compression algorithms

**FIGURE 3:**
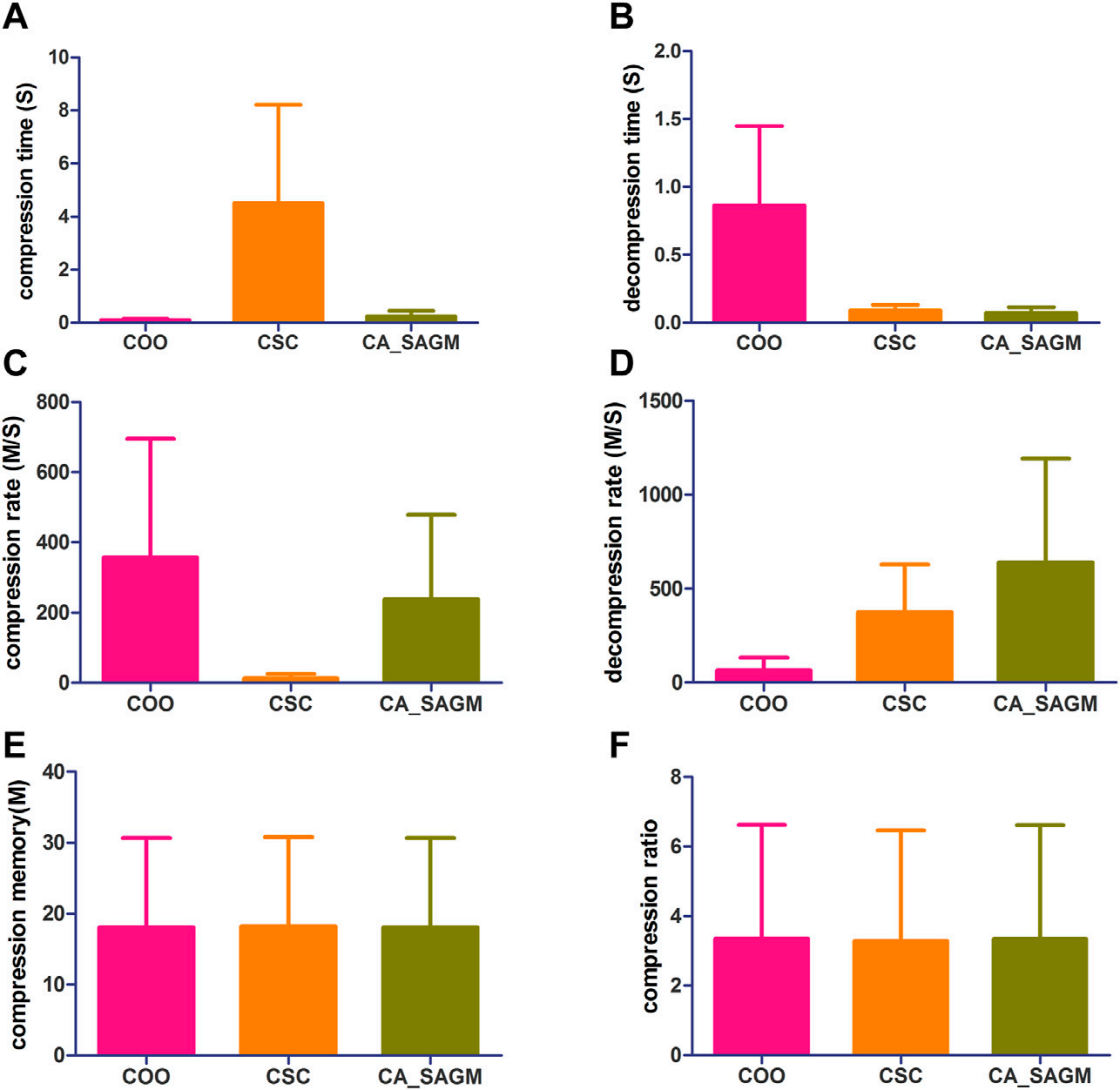
Compares the compression and decompression metrics of COO, CSC and CA_SAGM for CNV. Where **(A)** stands for compression time, **(B)** for decompression time, **(C)** for compression speed, **(D)** for decompression speed, **(E)** for compressed memory and **(F)** for compression ratio.

CNV data are more complex than SNV data, with larger datasets, a larger number of non-zeros and greater sparsity. Thus, we further investigated and analyzed the experimental results of the CNV data. Similarly, the process of processing CNV data includes steps such as data read-in, pre-processing, compression and storage. The raw CNV data is read in and tested for basic characteristics, including data set size (n), non-zero number (n), sparsity (%), rows (n), rows/columns (%), file size (K), L1-norm, L2-norm and rank. First, the CNV data runs the COO and CSC programs separately. The sparse data matrix was then preprocessed by row-first sorting and RCM sorting successively. Next, SNV data were run through CA_SAGM compression programs. Compression time, decompression time, compression rate, decompression rate, compression memory and compression ratio are respectively obtained by the three methods. The results are shown in Figure 3. Finally, the compressed data were stored in a suitable location. The experimental results were in mean ± SD, and were analyzed by comparing the evaluation indexes among different algorithms and using statistical methods.

From the Figure 3, we can see that in terms of compression performance, COO performs the best with the shortest CT (0.11 ± 0.06 vs. 4.51 ± 3.71 vs. 0.24 ± 0.21) and the largest CR (357.02 ± 337.97 vs. 12.72 ± 12.72 vs. 238.27 ± 240.35). CSC has the worst compression performance with the longest CT and the smallest CR. CA_SAGM had the middle compression performance. However, the CM (16.11 ± 12.45 vs. 16.11 ± 12.45 vs. 16.11 ± 12.45) and CRO (0.62 ± 0.41 vs. 0.62 ± 0.41 vs. 0.62 ± 0.41) were the same after compression by the three methods, which may be associated with a larger sparsity (19.58% ± 17.52%). In terms of decompression, COO had the worst performance, with the longest DCT (0.86 ± 0.59 vs. 0.09 ± 0.04 vs. 0.07 ± 0.04) and the smallest DCR (65.71 ± 67.19 vs. 375.74 ± 252.88 vs. 639.42 ± 553.6). CA_SAGM had the best decompression performance, with the shortest DCT and the smallest DCR. CSC decompression performance in the middle.

Similarly, a paired sample *t*-test was used to assess whether there were differences between any two algorithms for the same metrics. The results showed that almost all metrics were significantly different between any two algorithms (*p* < 0.05), with the exception of compression memory and compression ratio (*p* > 0.05). The detailed analysis results are as follows: Compression time (COO to CSC: 0.032; COO to CA_SAGM: 0.087; CA_SAGM to CSC: 0.031), decompression time (COO to CSC: 0.018; COO to CA_SAGM: 0.016; CA_SAGM to CSC: 0.000), compression rate (COO to CSC: 0.05; COO to CA_SAGM: 0.357; CA_SAGM to CSC: 0.06), decompression rate (COO to CSC: 0.011; COO to CA_SAGM: 0.034; CA_SAGM to CSC: 0.115), compression memory (COO to CSC: 0.018; COO to CA_SAGM: 0.002; CA_SAGM to CSC: 0.000), compression ratio (COO to CSC: 0.006; COO to CA_SAGM: 0.000; CA_SAGM to CSC: 0.007).

#### 3.2.2 Correlation analysis of CNV data

Spearman correlation analysis was used to investigate whether the compression and decompression performance was correlated with the basic characteristics of the CNV raw data (see Table 4). The results show that CT, DCT, CR, DCR, CM and CRO all have large correlation coefficients with the non-zero number, sparsity and L2-norm of the original data. In addition, CT, DCT and CM are strongly correlated with data file size and L1-norm. Also, there was a strong correlation between sparsity, non-zero number (*p* = 0.771) and L2-norm (*p* = 0.714). There was also a strong correlation between file size and L1-norm (*p* = 0.943).

**TABLE 4:** Spearman correlation analysis between compression and decompression metrics of COO, CSC and CA_SAGM algorithms for CNV data and basic characteristics of the original data.

Index	Data set size(n)	Non-zero number(n)	Sparsity (%)	Rows (n)	Row/column (%)	Non-negative ratio (%)	File size (K)	L1-norm	L2-norm	Rank
1_CT	−0.257	.943**	.829*	−0.257	−0.257	−0.143	0.771*	0.714*	.886*	0.143
2_CT	−0.029	1.000**	0.771*	−0.029	−0.029	0.086	.829*	0.771*	.943**	0.257
3_CT	0.257	.829*	0.543	0.257	0.257	−0.029	1.000**	.943**	.943**	0.6
1_DCT	−0.029	1.000**	0.771*	−0.029	−0.029	0.086	.829*	0.771*	.943**	0.257
2_DCT	−0.029	1.000**	0.771*	−0.029	−0.029	0.086	.829*	0.771*	.943**	0.257
3_DCT	−0.029	1.000**	0.771*	−0.029	−0.029	0.086	.829*	0.771*	.943**	0.257
1_CM	−0.029	1.000**	0.771*	−0.029	−0.029	0.086	.829*	0.771*	.943**	0.257
2_CM	−0.029	1.000**	0.771*	−0.029	−0.029	0.086	.829*	0.771*	.943**	0.257
3_CM	−0.029	1.000**	0.771*	−0.029	−0.029	0.086	.829*	0.771*	.943**	0.257
1_CR	0.6	−0.771*	−.886*	0.6	0.6	0.029	−0.429	−0.314	−0.657	0.314
2_CR	0.486	−.886*	−.943**	0.486	0.486	0.086	−0.6	−0.543	−0.771*	0.143
3_CR	0.257	−.943**	−.886*	0.257	0.257	−0.143	−0.657	−0.6	−.829*	0.029
1_DCR	0.6	−0.771*	−1.000**	0.6	0.6	−0.029	−0.543	−0.429	−0.714*	0.314
2_DCR	0.6	−0.771*	−1.000**	0.6	0.6	−0.029	−0.543	−0.429	−0.714*	0.314
3_DCR	0.6	−0.771*	−1.000**	0.6	0.6	−0.029	−0.543	−0.429	−0.714*	0.314
1_CRO	−0.6	0.771*	1.000**	−0.6	−0.6	0.029	0.543	0.429	0.714*	−0.314
2_CRO	−0.6	0.771*	1.000**	−0.6	−0.6	0.029	0.543	0.429	0.714*	−0.314
3_CRO	−0.6	0.771*	1.000**	−0.6	−0.6	0.029	0.543	0.429	0.714*	−0.314

Where 1_ represents the COO compression algorithm, 2_ represents the CSC compression algorithm and 3_ represents the CA_SAGM compression algorithm. ** At level 0.01, the correlation was significant.* At level 0.05, the correlation was significant.

**FIGURE 4:**
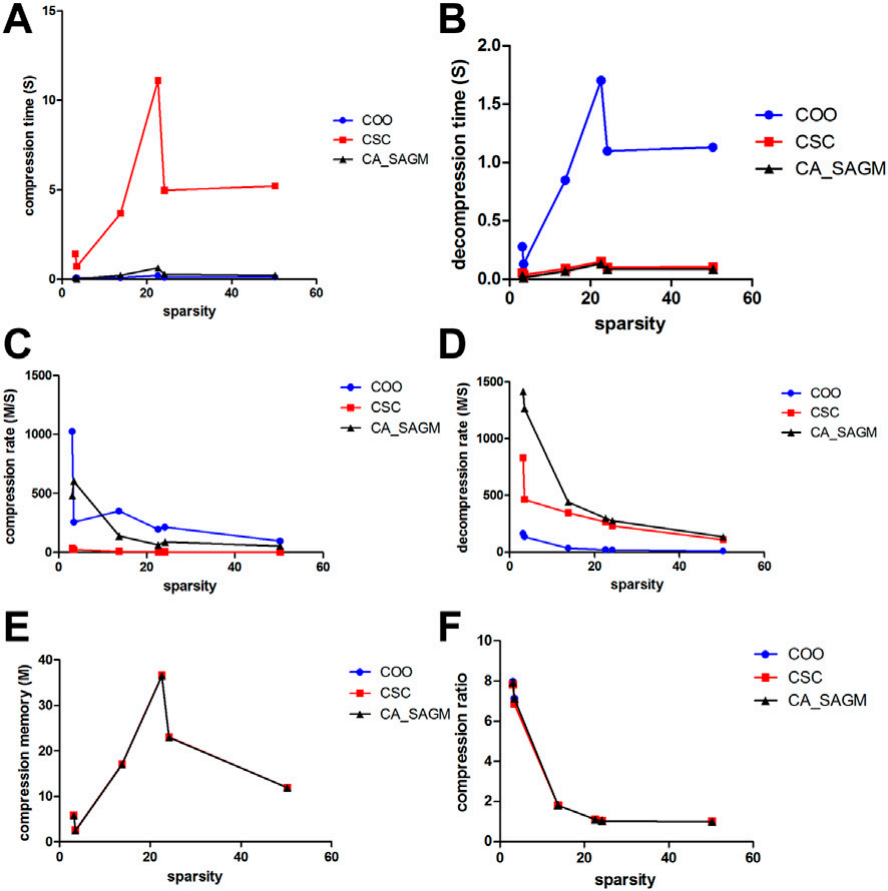
Curves of compression and decompression metrics vs. sparsity variation for COO, CSC and CA_SAGM for CNV. Where **(A)** stands for compression time, **(B)** for decompression time, **(C)** for compression speed, **(D)** for decompression speed, **(E)** for compressed memory and **(F)** for compression ratio.

Similarly, we have further analyzed the effect of the variation of CNV data sparsity on the experimental results, as shown in Figure 4. It can also be seen from the figure that in terms of compression performance, CSC has the worst compression characteristics, with the longest CT and the smallest CR. While both COO and CA_SAGM show better compression characteristics, with shorter CT and larger CR, with less difference between them. In terms of decompression, COO has the worst performance, with the longest DCT and the smallest DCR. CA_SAGM shows the best decompression characteristics, with the shortest DCT and the largest DCR. CSC decompression characteristics are between COO and CA_SAGM. When the sparsity is relatively small, the difference in compression and decompression performance between COO, CSC and CA_SAGM is small. The difference in compression and decompression time between CSC, COO and CA_SAGM increases as the sparsity increases. However, the difference between CR and DCR decreases with increasing sparsity.

## 4 Discussion and conclusion

In this paper, we propose a sparse asymmetric gene mutation compression algorithm CA_SAGM. The compression and decompression performance of COO, CSC and CA_SAGM is compared and analyzed using SNV and CNV data as the study objects. The results show that CA_SAGM can meet the high performance requirements of compression and decompression, achieve fast and lossless compression and decompression. In addition, it was found that the compression and decompression performance has a strong correlation with sparse. As the sparsity increases, all algorithms show longer compression and decompression times, lower compression and decompression rates, increased compression memory and lower compression ratios.

In our current study, CA_SAGM proved to have high compression and decompression performance for sparse genomic mutation data. CA_SAGM is a CSR compression algorithm for row-first sorting and reverse Cuthill-McKee sorting optimization. CA_SAGM has its own unique advantages over other compression algorithms. In combination with the reverse Cuthill-McKee sorting and optimization algorithm phase, the scattered non-zero elements of the data can be brought together on the diagonal and the bandwidth of the matrix is reduced considerably. Computational complexity versus memory and bandwidth based on the results of low-high (LU) decomposition. RCM pre-processing followed by LU decomposition can significantly reduce processing time, improve computational efficiency and reduce memory requirements. CA_SAGM has significant advantages in terms of compression and decompression time, as well as compression and decompression speed. CA_SAGM also has a very significant compression ratio advantage when the sparsity is low.

It should be noted that the results of this paper also have some limitations. Firstly, the SNV and CNV data from the experiments are limited and the sources of test data need to be expanded. Secondly, the data were only obtained from TCGA and the rest of the databases (e.g., GEO) were not studied. Recently, dedicated and integrated tools, genetic data compression algorithms, software and methods for compression in combination with machine learning (Wang et al., 2019; Kryukov et al., 2020; Chen et al., 2022; Niu et al., 2022; Yao et al., 2022) have received increasing attention and application by researchers, making it possible to process huge amounts of genetic data. For example, Cui Huanyu et al. proposed a new method of matrix compression based on CSR and COO: PBC algorithm for the problem that SPMV (sparse matrix vector multiplication) computation leads to computational redundancy, storage redundancy, load imbalance and low GPU utilization (Cui et al., 2022). The method considers load balancing conditions during the SPMV calculation. The blocks are divided according to a row-major order strategy, ensuring that the standard deviation between each block is minimized to satisfy the maximum similarity in the number of non-zero elements between each block. The result exhibits both speed-up ratio and compression performance. For lossless compression, researchers such as Jiabing Fu recommended LCQS; a lossless compression tool specialized for quality scores (Fu et al., 2020). The further development of specialized and integrated tools, software and evaluation methods, combined with artificial intelligence algorithms for the analysis and processing of genetic data are also the main directions and elements of our next research work. In summary, CA_SAGM has been shown to reduce data transfer time and storage space, and improve the utilization of network and storage resources. Promoting the use of this method will make the researcher’s work more effective and convenient.

## Data Availability

The original contributions presented in the study are included in the article/Supplementary Material, further inquiries can be directed to the corresponding authors.

## References

[B1] Ball M. P. Thakuria J. V. Zaranek A. W. Clegg T. Rosenbaum A. M. Wu X. (2012). A public resource facilitating clinical use of genomes. Proc. Natl. Acad. Sci. U. S. A. 109 (30), 11920–11927. 10.1073/pnas.1201904109 22797899 PMC3409785

[B2] Belsare S. Levy-Sakin M. Mostovoy Y. Durinck S. Chaudhuri S. Xiao M. (2019). Evaluating the quality of the 1000 genomes project data. Bmc Genomics 20 (1), 620. 10.1186/s12864-019-5957-x 31416423 PMC6696682

[B3] Boeke J. D. Church G. Hessel A. Kelley N. J. Arkin A. Cai Y. (2016). GENOME ENGINEERING. The genome project-write. Science 353 (6295), 126–127. 10.1126/science.aaf6850 27256881

[B4] Cavalli-Sforza L. L. (2005). The human genome diversity project: Past, present and future. Nat. Rev. Genet. 6 (4), 333–340. 10.1038/nrg1596 15803201

[B5] Chen D. Mao Y. Ding Q. Wang W. Zhu F. Chen C. (2020). Prognostic implications of programmed death ligand 1 expression in resected lung adenocarcinoma: A systematic review and meta-analysis. Eur. J. Cardio-Thoracic Surg. 58 (5), 888–898. 10.1093/ejcts/ezaa172 32596715

[B6] Chen H. Chen J. Lu Z. Wang R. (2022). Cmic: An efficient quality score compressor with random access functionality. BMC Bioinforma. 23 (1), 294. 10.1186/s12859-022-04837-1 PMC9308261 35870880

[B7] Chen X. Xie P. Chi L. Liu J. Gong C. (2018). An efficient SIMD compression format for sparse matrix-vector multiplication. Concurrency Computation-Practice Exp. 30 (23), e4800. 10.1002/cpe.4800

[B8] Cui H. Wang N. Wang Y. Han Q. Xu Y. (2022). An effective SPMV based on block strategy and hybrid compression on GPU. J. Supercomput. 78 (5), 6318–6339. 10.1007/s11227-021-04123-6

[B9] Fairley S. Lowy-Gallego E. Perry E. Flicek P. (2020). The International Genome Sample Resource (IGSR) collection of open human genomic variation resources. Nucleic Acids Res. 48 (D1), D941–D947. 10.1093/nar/gkz836 31584097 PMC6943028

[B10] Fira C. M. Goras L. (2008). An ECG signals compression method and its validation using NNs. Ieee Trans. Biomed. Eng. 55 (4), 1319–1326. 10.1109/TBME.2008.918465 18390322

[B11] Fu J. Ke B. Dong S. (2020). Lcqs: An efficient lossless compression tool of quality scores with random access functionality. BMC Bioinforma. 21 (1), 109. 10.1186/s12859-020-3428-7 PMC7079445 32183707

[B12] Garand M. Kumar M. Huang S. S. Y. Al Khodor S. (2020). A literature-based approach for curating gene signatures in multifaceted diseases. J. Transl. Med. 18 (1), 279. 10.1186/s12967-020-02408-7 32650786 PMC7350750

[B13] Huang T. Li J. Jia B. Sang H. (2021). CNV-MEANN: A neural network and mind evolutionary algorithm-based detection of copy number variations from next-generation sequencing data. Front. Genet. 12, 700874–708021. 10.3389/fgene.2021.700874 34484298 PMC8415314

[B14] Janssen S. Ramaswami G. Davis E. E. Hurd T. Airik R. Kasanuki J. M. (2011). Mutation analysis in Bardet-Biedl syndrome by DNA pooling and massively parallel resequencing in 105 individuals. Hum. Genet. 129 (1), 79–90. 10.1007/s00439-010-0902-8 21052717 PMC3646619

[B15] Jugas R. Sedlar K. Vitek M. Nykrynova M. Barton V. Bezdicek M. (2021). CNproScan: Hybrid CNV detection for bacterial genomes. Genomics 113 (5), 3103–3111. 10.1016/j.ygeno.2021.06.040 34224809

[B16] Kim M. J. Lee S. Yun H. Cho S. I. Kim B. Lee J. S. (2022). Consistent count region-copy number variation (CCR-CNV): An expandable and robust tool for clinical diagnosis of copy number variation at the exon level using next-generation sequencing data. Genet. Med. 24 (3), 663–672. 10.1016/j.gim.2021.10.025 34906491

[B17] Koza Z. Matyka M. Szkoda S. Mirosław Ł. (2014). Compressed multirow storage format for sparse matrices on graphics processing units. Siam J. Sci. Comput. 36 (2), C219–C239. 10.1137/120900216

[B18] Kryukov K. Ueda M. T. Nakagawa S. Imanishi T. (2020). Sequence Compression Benchmark (SCB) database-A comprehensive evaluation of reference-free compressors for FASTA-formatted sequences. Gigascience 9 (7), giaa072. 10.1093/gigascience/giaa072 32627830 PMC7336184

[B19] Ladeira G. C. Pilonetto F. Fernandes A. C. Bóscollo P. P. Dauria B. D. Titto C. G. (2022). CNV detection and their association with growth, efficiency and carcass traits in Santa Ines sheep. J. Animal Breed. Genet. 139 (4), 476–487. 10.1111/jbg.12671 35218589

[B20] Lavrichenko K. Johansson S. Jonassen I. (2021). Comprehensive characterization of copy number variation (CNV) called from array, long- and short-read data. BMC Genomics 22 (1), 826. 10.1186/s12864-021-08082-3 34789167 PMC8596897

[B21] Lee W.-P. Zhu Q. Yang X. Liu S. Cerveria E. Ryan M. (2022). A whole-genome sequencing-based algorithm for copy number detection at clinical grade level. Genomics, proteomics Bioinforma. 20. 1197. 10.1016/j.gpb.2021.06.003 PMC10225484 35085778

[B22] Lewin H. A. Robinson G. E. Kress W. J. Baker W. J. Coddington J. Crandall K. A. (2018). Earth BioGenome project: Sequencing life for the future of life. Proc. Natl. Acad. Sci. U. S. A. 115 (17), 4325–4333. 10.1073/pnas.1720115115 29686065 PMC5924910

[B23] Li B. Yu L. Gao L. (2022). Cancer classification based on multiple dimensions: SNV patterns. Comput. Biol. Med. 151, 106270. 10.1016/j.compbiomed.2022.106270 36395594

[B24] Li R. Chang C. Tanigawa Y. Narasimhan B. Hastie T. Tibshirani R. (2021). Fast numerical optimization for genome sequencing data in population biobanks. Bioinformatics 37 (22), 4148–4155. 10.1093/bioinformatics/btab452 34146108 PMC9206591

[B25] Macintyre G. Ylstra B. Brenton J. D. (2016). Sequencing structural variants in cancer for precision therapeutics. Trends Genet. 32 (9), 530–542. 10.1016/j.tig.2016.07.002 27478068

[B26] Medvedev P. Stanciu M. Brudno M. (2009). Computational methods for discovering structural variation with next-generation sequencing. Nat. Methods 6 (11), S13–S20. 10.1038/nmeth.1374 19844226

[B27] Moffat A. (2019). Huffman coding. Acm Comput. Surv. 52 (4), 1–35. 10.1145/3342555

[B28] Mota N. R. Franke B. (2020). 30-year journey from the start of the human genome project to clinical application of genomics in psychiatry: Are we there yet? Lancet Psychiatry 7 (1), 7–9. 10.1016/S2215-0366(19)30477-8 31860458

[B29] Naqvi S. Naqvi R. Riaz R. R. Siddiqi F. (2011). Optimized RTL design and implementation of LZW algorithm for high bandwidth applications. Przeglad Elektrotechniczny 87 (4), 279–285.

[B30] Niu Y. Ma M. Li F. Liu X. Shi G. (2022). ACO:lossless quality score compression based on adaptive coding order. BMC Bioinforma. 23 (1), 219. 10.1186/s12859-022-04712-z PMC9175485 35672665

[B31] Oh S. Lee J. Kwon M. S. Weir B. Ha K. Park T. (2012). A novel method to identify high order gene-gene interactions in genome-wide association studies: Gene-based MDR. Bmc Bioinforma. 13, S5. 10.1186/1471-2105-13-S9-S5 PMC3372457 22901090

[B32] Park J. Yi W. Ahn D. Kung J. Kim J. J. (2020). Balancing computation loads and optimizing input vector loading in LSTM accelerators. Ieee Trans. Computer-Aided Des. Integr. Circuits Syst. 39 (9), 1889–1901. 10.1109/tcad.2019.2926482

[B33] Prashant N. M. Liu H. Dillard C. Ibeawuchi H. Alsaeedy T. Chan H. (2021). Improved SNV discovery in barcode-stratified scRNA-seq alignments. Genes 12 (10), 1558. 10.3390/genes12101558 34680953 PMC8535975

[B34] Press M. O. Hall A. N. Morton E. A. Queitsch C. (2019). Substitutions are boring: Some arguments about parallel mutations and high mutation rates. Trends Genet. 35 (4), 253–264. 10.1016/j.tig.2019.01.002 30797597 PMC6435258

[B35] Samaha G. Wade C. M. Mazrier H. Grueber C. E. Haase B. (2021). Exploiting genomic synteny in felidae: Cross-species genome alignments and SNV discovery can aid conservation management. Bmc Genomics 22 (1), 601. 10.1186/s12864-021-07899-2 34362297 PMC8348863

[B36] Schnepp P. M. Chen M. Keller E. T. Zhou X. (2019). SNV identification from single-cell RNA sequencing data. Hum. Mol. Genet. 28 (21), 3569–3583. 10.1093/hmg/ddz207 31504520 PMC7279618

[B37] Shekaramiz M. Moon T. K. Gunther J. H. (2019). Bayesian compressive sensing of sparse signals with unknown clustering patterns. Entropy 21 (3), 247. 10.3390/e21030247 33266961 PMC7514728

[B38] Stankiewicz P. Lupski J. R. (2010). Structural variation in the human genome and its role in disease. Annu. Rev. Med. 61, 437–455. 10.1146/annurev-med-100708-204735 20059347

[B39] The ICGC/TCGA Pan-Cancer Analysis of Whole Genomes Consortium (2020). Pan-cancer analysis of whole genomes. Nature 578 (7793), 82. 32025007 10.1038/s41586-020-1969-6 PMC7025898

[B40] Tu Z. D. Wang L. Xu M. Zhou X. Chen T. Sun F. (2006). Further understanding human disease genes by comparing with housekeeping genes and other genes. Bmc Genomics 7, 31. 10.1186/1471-2164-7-31 16504025 PMC1397819

[B41] van der Borght K. Thys K. Wetzels Y. Clement L. Verbist B. Reumers J. (2015). QQ-SNV: Single nucleotide variant detection at low frequency by comparing the quality quantiles. Bmc Bioinforma. 16, 379. 10.1186/s12859-015-0812-9 PMC4641353 26554718

[B42] Wang J. Ding D. Li Z. Feng X. Cao C. Ma Z. (2022). Sparse tensor-based multiscale representation for point cloud geometry compression. IEEE Trans. pattern analysis Mach. Intell. 2022, 1. 10.1109/TPAMI.2022.3225816 36455091

[B43] Wang R. Zang T. Wang Y. (2019). Human mitochondrial genome compression using machine learning techniques. Hum. Genomics 13 (1), 49. 10.1186/s40246-019-0225-3 31639043 PMC6805717

[B44] Xi J. Deng Z. Liu Y. Wang Q. Shi W. (2023). Integrating multi-type aberrations from DNA and RNA through dynamic mapping gene space for subtype-specific breast cancer driver discovery. Peerj 11, e14843. 10.7717/peerj.14843 36755866 PMC9901305

[B45] Xi J. Li A. (2016). Discovering recurrent copy number aberrations in complex patterns via non-negative sparse singular value decomposition. Ieee-Acm Trans. Comput. Biol. Bioinforma. 13 (4), 656–668. 10.1109/TCBB.2015.2474404 26372614

[B46] Xi J. Li A. Wang M. (2020). HetRCNA: A novel method to identify recurrent copy number alternations from heterogeneous tumor samples based on matrix decomposition framework. Ieee-Acm Trans. Comput. Biol. Bioinforma. 17 (2), 422–434. 10.1109/TCBB.2018.2846599 29994262

[B47] Xi J. Sun D. Chang C. Zhou S. Huang Q. (2023). An omics-to-omics joint knowledge association subtensor model for radiogenomics cross-modal modules from genomics and ultrasonic images of breast cancers. Comput. Biol. Med. 155, 106672. 10.1016/j.compbiomed.2023.106672 36805226

[B48] Xi J. Yuan X. Wang M. Li X. Huang Q. (2020). Inferring subgroup-specific driver genes from heterogeneous cancer samples via subspace learning with subgroup indication. Bioinformatics 36 (6), 1855–1863. 10.1093/bioinformatics/btz793 31626284

[B49] Xing L. Wang Z. Ding Z. Chu G. Dong L. Xiao N. (2022). An efficient sparse stiffness matrix vector multiplication using compressed sparse row storage format on AMD GPU. Concurrency Computation-Practice Exp. 34 (23). 10.1002/cpe.7186

[B50] Yao H. Hu G. Liu S. Fang H. Ji Y. (2022). SparkGC: Spark based genome compression for large collections of genomes. BMC Bioinforma. 23 (1), 297. 10.1186/s12859-022-04825-5 PMC9310413 35879669

[B51] Yao W. Huang F. Zhang X. Tang J. (2019). Ecogems: Efficient compression and retrieve of SNP data of 2058 rice accessions with integer sparse matrices. Bioinformatics 35 (20), 4181–4183. 10.1093/bioinformatics/btz186 30873546

[B52] Zheng T. (2022). DETexT: An SNV detection enhancement for low read depth by integrating mutational signatures into TextCNN. Front. Genet. 13, 943972–948021. (Print)). 10.3389/fgene.2022.943972 36246660 PMC9554618

